# The Spectrum and Temporal Trends of Glomerular Diseases in Saudi Arabia: A Systematic Review of Biopsy‐Based Studies

**DOI:** 10.1155/ijne/3670670

**Published:** 2026-04-23

**Authors:** Manal E. Alotaibi, Laila A. Alharbi, Fahd Almalki, Waleed Alotaibi, Ibrahim Tawhari, Mutlaq Alotaibi

**Affiliations:** ^1^ Department of Medicine, College of Medicine, Umm Al-Qura University, Makkah, Saudi Arabia, uqu.edu.sa; ^2^ Department of Internal Medicine, College of Medicine, King Khalid University, Abha, Saudi Arabia, kku.edu.sa; ^3^ Department of Nephrology and Kidney Transplantation, AlHada Armed Forces Hospital, Taif, Saudi Arabia

**Keywords:** epidemiology, glomerular diseases, glomerulonephritis, nephropathy, prevalence, renal biopsy, Saudi Arabia

## Abstract

**Purpose:**

This systematic review aims to synthesize data from biopsy‐based studies to quantify the epidemiology, regional variations, and temporal trends of glomerular diseases (GDs) in Saudi Arabia.

**Methods:**

We conducted a systematic search using PubMed, MEDLINE, the Cochrane Library, and Google Scholar to identify relevant studies published up to January 1, 2025. Studies reporting native renal‐biopsy data from Saudi Arabia, with histopathological confirmation of GD, were included. Data extraction followed Preferred Reporting Items for Systematic Reviews and Meta‐Analyses (PRISMA) guidelines, and study quality was assessed using the Newcastle–Ottawa Scale.

**Results:**

Twelve studies (4066 patients) were included, representing all major regions of Saudi Arabia between 1989 and 2017. Primary GD accounted for 50%–80% of all biopsies, while secondary GD represented 18%–48%. Focal segmental glomerulosclerosis (FSGS) was the most common primary GD (15%–40% of cases), while lupus nephritis dominated secondary GD (55%–75%). Nephrotic syndrome was the principal indication for biopsy in all adult cohorts (40%–65%), particularly with minimal change disease (MCD), FSGS, and membranous glomerulonephritis (MGN) subtypes. Temporal trends revealed an increase in IgA nephropathy and diabetic nephropathy, with a decline in membranoproliferative glomerulonephritis. Notable regional variations were also identified. Progression to ESRD was observed in 25%–35% of crescentic and FSGS cases.

**Conclusion:**

This review highlights the evolving landscape of GDs in Saudi Arabia, emphasizing the need for early diagnosis, standardized biopsy protocols, and multidisciplinary management. A national renal biopsy registry is recommended to improve surveillance and research collaboration.

## 1. Introduction

Glomerular diseases (GDs) represent a diverse group of disorders characterized by inflammation, sclerosis, or damage to the glomeruli [1]. These conditions encompass both primary glomerulonephritis, where the kidney is the primary target organ, and secondary forms that occur as manifestations of systemic diseases such as diabetes mellitus, systemic lupus erythematosus, or infectious processes [2]. The classification of GDs has evolved significantly, with current approaches emphasizing immunopathogenesis rather than purely histological patterns [3]. The pathophysiology of GDs involves complex interactions between immune mechanisms, complement activation, and cellular responses within the glomerular architecture, manifesting clinically as proteinuria, hematuria, hypertension, and progressive decline in kidney function [4].

The clinical importance and healthcare burden of GDs arise not only from the complexities of their management but also from their strong association with cardiovascular complications and their potential to progress to chronic kidney disease (CKD) and end‐stage kidney failure (ESKD) [5]. This dual impact compromises patient quality of life and imposes substantial demands on healthcare systems bearing the costs of dialysis, kidney transplantation, and management of associated complications [6].

Global epidemiological data demonstrate significant geographical and ethnic variations in the prevalence and patterns of GDs. In North America, diabetic glomerulosclerosis (GS) and focal segmental glomerulosclerosis (FSGS) are the most predominant diagnoses, each accounting for 19.1% of all GDs. In Latin America, lupus nephritis is the most common, representing a significant 38.1% of cases, followed by FSGS at 15.8%. In Europe, IgA nephropathy (IgAN) is the leading diagnosis at 22.1%, with FSGS being the second most frequent at 14.9%. In Asia, IgAN is markedly predominant, accounting for 39.5% of GDs, followed by lupus nephritis at 16.8% [7]. These variations reflect the multifaceted interplay of genetic susceptibility, environmental factors, infectious exposures, and diagnostic standards across different regions [8].

Several studies have reported on GDs prevalence in Saudi Arabia, but findings vary due to regional differences, small sample sizes, and evolving diagnostic criteria. FSGS has consistently been identified as the predominant primary GD, with rates notably higher than those observed in many Asian countries. The association between FSGS and obesity is another significant finding in the Saudi Arabian context, where obesity prevalence has reached up to 39% in the adult population [9, 10].

Secondary GDs in Saudi Arabia are primarily dominated by lupus nephritis, reflecting the increased susceptibility of certain populations to autoimmune diseases and aligning with global trends. Correspondingly, there has been a notable rise in diabetic nephropathy as a cause of secondary GD [10]. This sharp increase parallels the growing prevalence of type 2 diabetes mellitus in Saudi Arabia, which has reached epidemic levels, with rates exceeding 28% in the adult population [11].

The Kingdom of Saudi Arabia has undergone rapid socioeconomic development and urbanization over the past several decades accompanied by significant changes in lifestyle, dietary habits, and disease prevalence. While these transformations are expected to remodel the GDs landscape, previous studies have primarily been single‐center, retrospective, and limited in scope, making it difficult to ascertain temporal trends and regional differences. This systematic review synthesizes three decades of data to quantify the epidemiology and regional variations of GDs in Saudi Arabia, assess temporal changes in disease trends, and compare the findings with global literature. By consolidating existing evidence, the review is expected to support local nephrologists in their diagnostic and therapeutic practices, inform public health policies, and identify research gaps for future studies.

## 2. Materials and Methods

This review systematically compiles data from published peer‐reviewed studies on GDs in Saudi Arabia. It covers biopsy‐proven cases in both adults and children.

### 2.1. Search Strategy

We conducted a systematic search using PubMed, MEDLINE, the Cochrane Library, and Google Scholar to identify relevant studies available as of January 01, 2025. The search strategy was developed using a combination of Medical Subject Headings (MeSH) terms and Boolean operators (AND, OR) to ensure comprehensive retrieval of studies. Keywords were selected based on their relevance to GDs and were systematically categorized as outlined in Table S1 of the Supporting Information.

### 2.2. Study Selection Criteria

**FIGURE 1:**
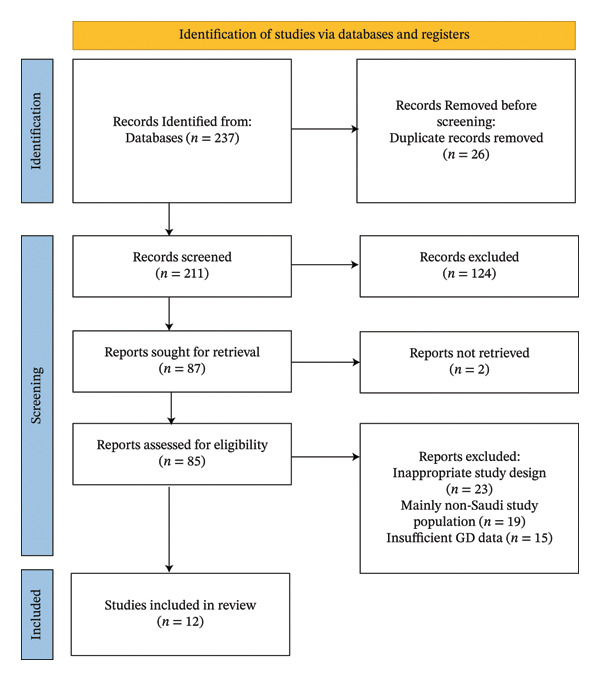
PRISMA study identification and selection flowchart.

The study selection process is outlined in Figure 1. We followed the Preferred Reporting Items for Systematic Reviews and Meta‐Analyses (PRISMA) guidelines for study selection [12]. The initial search yielded a broad range of articles, which were subsequently screened for eligibility based on predefined inclusion and exclusion criteria.

#### 2.2.1. Inclusion Criteria

• Studies reporting native renal‐biopsy data from any region of Saudi Arabia.
• Epidemiological, cross‐sectional, and cohort studies published in peer‐reviewed journals.
• Studies conducted on human subjects with histopathological confirmation of GD.
• Articles written in English or with an available translated version in English.

#### 2.2.2. Exclusion Criteria

• Case reports, case–control studies, review articles, and editorials.
• Studies primarily involving non‐Saudi populations or lacking specific regional data.
• Articles with insufficient or unclear methodology regarding disease classification.

### 2.3. Data Extraction

Two researchers independently screened and extracted data from the included studies using a structured form. They then cross‐verified the extracted results and resolved any discrepancies. The extracted data included study design, sample size, patient demographics, GD subtypes, comorbidities, presenting syndromes, temporal changes, and follow‐up outcomes.

### 2.4. Synthesis Approach

A narrative synthesis was conducted to summarize the evidence due to the significant clinical and methodological heterogeneity across the included observational studies. This process involved a textual summary of the findings to build a coherent narrative that highlights both consistencies and discrepancies. This evidence‐focused synthesis rather than an epidemiologically quantitative approach reflects the descriptive nature of the available data. No meta‐analysis of effect size was attempted because all included reports were observational and lacked comparison arms; additionally, there was a potential overlap of patient populations across some studies.

### 2.5. Quality Assessment

The quality of the included studies was assessed using the Newcastle–Ottawa Scale (NOS) [13], a widely utilized tool for evaluating observational studies. The NOS assigns one or two points across eight items, organized into three domains: selection of study groups, comparability of groups, and outcome ascertainment. Scores range from 0 to 9 stars, with 7–9 indicating high‐quality studies, 5‐6 representing moderate quality, and 0–4 denoting low quality.

## 3. Results

### 3.1. Overview of the Included Studies

A total of 12 studies met inclusion criteria, collectively representing 4066 patients who underwent renal biopsies across various regions of Saudi Arabia between 1989 and 2017 [10, 14–24]. The studies comprised nine single‐center retrospective analyses, two retrospective multicenter collaborations, and one multicenter registry‐based study. The studies represented all major geographical regions of Saudi Arabia: Central (*n* = 5, 41.7%), Western (*n* = 3, 25%), Eastern (*n* = 2, 16.7%), and multicenter studies covering multiple regions (*n* = 2, 16.7%).

The included studies varied considerably in size, ranging from 19 biopsies [21] to 1760 biopsies [10]. Most studies (87.5%) utilized all three diagnostic modalities (light microscopy, immunofluorescence, and electron microscopy), though availability of electron microscopy was limited in some centers, particularly in earlier time periods. The key findings and characteristics of the included studies are presented in Table 1.

**TABLE 1:** Summary of included studies on glomerular diseases in Saudi Arabia.

Study & year	Study design	Study setting	Sample size	Primary GN, *n* (%)	Secondary GN, *n* (%)	Diagnostic tests used	Key findings
[14] 1996	Single‐center, Retrospective	Riyadh (King Khalid University Hospital)	186 adults	147 (79%)	33 (17.7%)	LM IF EM (Selected cases)	GD is characterized by a high frequency of FSGS, which is a leading cause of nephrotic syndrome.

[15] 2000	Multicenter Registry	Riyadh (King Khalid University Hospital, Riyadh Kharj Military Hospital, King Fahed National Guard Hospital and Riyadh Medical Complex). Jeddah (Kidney Center) and the University Hospital from the eastern province (King Faisal University Hospital)	782 adults	587 (72.6%)	221 (27.4%)	LM IF EM	FSGS and MPGN were the most common forms of primary GN in adult patients. LN was the most common cause of secondary GN.

[16] 2010	Single‐center, Retrospective	Al‐Khobar (King Fahd Hospital of the University)	233 (adults & children)	187 (80.3%)	46 (19.7%)	LM (All cases) IF (149 cases) EM (34 cases)	MCG and LN were the most prevalent primary and secondary GN, respectively, in both pediatric and adult age groups, as well as in both males and females in the Eastern Province.

[17] 2013	Single‐center, Retrospective	Riyadh (Armed Forces Hospital)	348 (adults & children)	192 (55.1%)	132 (37.9%)	LM IF EM	The pattern of GN seems to vary in different regions of Saudi Arabia.

[18] 2014	Single‐center (Jeddah), Retrospective	Jeddah (King Abdulaziz University)	103 with MesPGN	N/A^a^	N/A	LM IF EM	MesPGN is an important cause of nephrotic syndrome in young adults in the western region of Saudi Arabia, with IgM Nephropathy being its most common underlying cause.

[19] 2017	Single‐center, Retrospective	Riyadh (King Faisal Specialist Hospital)	78 with crescentic GN	N/A^b^	N/A	LM IF EM	Most cases of GN with crescents were due to immune complex–mediated GN, followed by pauci‐immune GN and anti‐GBM GN. LN was the most common cause of immune complex–mediated crescentic GN.

[20] 2017	Single‐center, Retrospective	Riyadh (King Fahad Medical City)	166 (age ≥ 12)	102 (61.4%)	64 (38.6%)	LM IF EM	

[21] 2018	Single‐center (Jeddah), Retrospective	Jeddah (King Abdulaziz University Hospital)	19 children	15 (78.9%)	4 (21.1%)	LM IF EM	PIGN was the most common etiology of RPGN in the pediatric cohort, and these patients achieved a good clinical prognosis overall.

[22] 2019	Single‐center (Madinah), Retrospective	Madina (King Fahd Hospital)	44	23 (52.3%)	21 (47.7%)	LM IF EM in one case only	Membranous nephropathy was found to be the predominant primary glomerular disease.

[10] 2019	Multicenter, Retrospective	Riyadh (King Saud University Medical City, King Faisal Specialist Hospital, Security Forces Hospital, and Research Center, King Abdulaziz Medical City) Jeddah (King Abdulaziz University Hospital)	1070 adults	535 (50%)	407 (38%)	LM IF EM	While FSGS remains the most common GD, trends in biopsy‐diagnosed glomerular disease have changed, with a significant rise in the prevalence of IgAN and Diabetic Nephropathy, and a decline in MPGN.

[23] 2020	Multicenter, Retrospective	Riyadh (King Saud University Medical City, Security Forces Hospital, King Abdulaziz Medical City) Jeddah (King Abdulaziz University Hospital)	326 children	182 (55.8%)	138 (42.3%)	LM IF EM	There was a considerable shift in the frequency of many GD subtypes in children between 1998 and 2017, with a notable decline in MPGN and MesPGN. LN is the most common cause of secondary GN.

[24] 2020	Single‐center, Retrospective	Jeddah (King Abdulaziz University Hospital)	711 adults	448 (63%)	263 (37%)	LM IF	The western region of Saudi Arabia presents with a different primary GN pattern than the rest of the country, likely due to its unique geographical and environmental characteristics.

*Note:* FSGS, focal segmental glomerulosclerosis; IF, immunofluorescence; MPGN, membranoproliferative glomerulonephritis; RPGN, rapidly progressive glomerulonephritis.

Abbreviations: EM, electron microscopy; LM, light microscopy; LN, lupus nephritis; MCD, minimal change disease; MN, membranous nephropathy; NA, not available.

^a^IgM nephropathy: 47%, IgA nephropathy: 30%, lupus nephritis (Class II): 9%, FSGS: 5%.

^b^Immune complex–mediated GN: 71.8% (of which lupus nephritis was the most common cause), Pauci‐immune GN: 20.5%, Anti‐GBM GN: 7.7%.

### 3.2. Quality Assessment of the Included Studies

The majority of the included studies were of high quality, scoring 7–8 stars on the NOS. All studies were based on biopsy‐proven diagnoses with standardized pathology reviews. They typically featured well‐defined populations, either from multicenter collaborations or single‐center tertiary referral centers, with clear inclusion and exclusion criteria. Several studies also had large sample sizes. Comparability was further enhanced in multicenter and registry‐based studies, which accounted for confounders like age, sex, and region.

However, there were notable limitations. The retrospective design of nearly all studies introduced risks such as missing data, selection bias, and limited follow‐up for outcomes. Some single‐center studies had small sample sizes or exhibited regional referral bias, as seen in studies by Al‐Hussiain et al. [19], Manzoor et al. [22], and Mosaad et al. [21]. Pediatric cohorts were particularly underrepresented, with smaller sample sizes in studies focusing on this population. The results of the quality assessment are summarized in the Supporting Information Table S2.

### 3.3. Distribution and Clinical Presentation of GDs

Primary GN accounted for 50%–80% of all biopsies, while secondary GN represented 18%–48%. The relative frequencies of primary GN subtypes show considerable variation across studies, influenced by region, time period, and patient population (adult vs. pediatric). Across most large‐scale adult studies, FSGS was the most frequently diagnosed primary GN, with prevalence rates ranging from 15% to 40%. It was also the leading cause in the pediatric population, alongside MCD (Table 2).

**TABLE 2:** Prevalence of major primary glomerulonephritis subtypes in Saudi Arabia (% of all primary GN cases).

Study (author, year)	FSGS	MCD	MN	IgAN	MPGN	MesPGN	Total primary GN
Mitwalli et al., 1996 [14]	40.8% (60)	1.4% (2)	13.6% (20)	13.6% (20)	9.5% (14)	21.1% (31)	147
Huraib et al., 2000 [15]	21.3% (125)	11.6% (68)	10.6% (62)	6.5% (38)	20.7% (122)	16.3% (96)	587
Shawarby et al., 2010 [16]	15.5% (29)	29.4% (55)	8.6% (16)	6.4% (12)	9.6% (18)	19.8% (37)	187
Aslam et al., 2013 [17]	27.6% (53)	17.7% (34)	9.9% (19)	11.5% (22)	13.0% (25)	12.5% (24)	192
AlMatham et al, 2017 [20]	35.3% (36)	26.5% (27)	12.7% (13)	7.8% (8)	5.9% (6)	—	102
Manzoor et al, 2019 [22]	17.4% (4)	8.7% (2)	39.1% (9)	8.7% (2)	—	4.3% (1)	23
AlFaadhel et al., 2019 [10]	39.8% (213)	6.9% (37)	17.0% (91)	29.2% (156)	7.1% (38)	—	535
Alhasan et al., 2020 [23]	30.8% (56)	28.6% (52)	7.2% (13)	9.3% (17)	12.6% (23)	11.5% (21)	182
Vachharajani et al., 2021 [24]	22.5% (101)	8.0% (36)	24.1% (108)	20.3% (91)	9.8% (44)	—	448

*Note:* FSGS, focal segmental glomerulosclerosis; MesPGN, mesangioproliferative glomerulonephritis; MPGN, membranoproliferative glomerulonephritis.

Abbreviations: IgAN, IgA nephropathy; MCD, minimal change disease; MN, membranous nephropathy.

A long‐term study by Shawarby et al. found MCD to be the most common primary GN (29.4%), a finding that differs from most other reports in the Kingdom [16]. Manzoor et al. also reported that membranous nephropathy was the most common primary disease, accounting for 20.45% [22].

Lupus nephritis was the most common secondary GN (55%–75%), with Class IV being the predominant subtype. Amyloidosis (3%–6%), ANCA vasculitis (≈2%), and Alport syndrome (< 1%) were consistently rare (Table 3).

**TABLE 3:** Secondary glomerular diseases frequency.

Disease	Frequency (%)	Notes
Lupus nephritis	25–75	Most common secondary, esp. females
Diabetic nephropathy	1–10	DM prevalence increasing, rising
Amyloidosis	< 5	Rarer than regional neighbors
Hypertensive	< 5	Less common, older patients
Crescentic GN	2–7	SLE, PIGN, ANCA, anti‐GBM as causes

*Note:* ANCA, antineutrophil cytoplasmic antibody; GN, glomerulonephritis; PIGN, post‐infectious glomerulonephritis.

Abbreviations: Anti‐GBM, anti‐glomerular basement membrane; LN, lupus nephritis; SLE, systemic lupus erythematosus.

Nephrotic syndrome was the principal indication for biopsy in all adult cohorts (40%–65%), particularly with MCD, FSGS, and MGN subtypes. Unexplained renal impairment and nephritic syndrome predominated in IgAN, MPGN, and pauci‐immune crescentic GN. Other common presentations include unexplained renal impairment, proteinuria, and hematuria. In pediatrics, macroscopic hematuria followed upper‐respiratory infection dominated [21].

Elevated serum creatinine at presentation was frequently observed in cases of crescentic GN, and was a strong predictor of poor renal outcomes. Heavy proteinuria (> 3 g/day) was common in FSGS, MGN, and MCD, whereas microscopic hematuria was universally present in IgAN and post‐infectious glomerulonephritis (PIGN). Additionally, serological findings played a crucial role in several diagnoses: low complement levels (C3) were characteristic of PIGN and MPGN, while ANCA was vital for diagnosing pauci‐immune vasculitis [21].

Some studies focused on specific pathological, morphological, or rapidly progressive presentations. A study from the Western region found that among patients with a MesPGN pattern on light microscopy, the most common final diagnosis after immunofluorescence and electron microscopy was IgM nephropathy (46.6%), followed by IgAN (30%).

Studies focusing on the rapidly progressive glomerulonephritis (RPGN) severe presentation show that immune complex–mediated GN is the most common underlying cause in adults, with LN being the single most frequent etiology. In children, however, RPGN is most commonly caused by PIGN.

### 3.4. Patient Demographics and Comorbidities

Across the studies focusing on adults, the mean age of patients undergoing renal biopsy for GD was typically in the third and fourth decades of life. Pediatric studies showed a mean age of diagnosis around 8.5–11 years.

In adult primary GN, there is a consistent male predominance, with male‐to‐female ratios ranging from 1.3:1 to as high as 6:1 for IgAN. Conversely, secondary GN, driven by the high prevalence of LN, shows a strong female predominance. In the pediatric population, a male predominance was also noted for most primary GNs.

Regarding comorbidities, hypertension was documented in 40% or more of cases at presentation. Systemic lupus erythematosus was observed in 23%–26% of adult biopsies [10, 15]. Diabetes mellitus was present in 12%–18% at the time of biopsy, with diabetic nephropathy primarily biopsied for atypical presentations [10, 20].

### 3.5. Temporal Changes in GD

Several studies, particularly the large‐scale analyses by AlFaadhel et al. [10] and Jalalah [24], have highlighted significant shifts in the patterns of GDs over the last two to three decades. While FSGS remains the most common primary GD (35.1% in recent years), there has been a dramatic rise in IgAN, increasing from 9.1% to 22.8%. This makes IgAN the second most common primary GN in recent large‐scale adult studies, nearly matching FSGS in prevalence. Conversely, there has been a precipitous decline in MPGN, dropping from 15.4% to 8.4%, as well as a decrease in MCD among adults over time.

In secondary GN, LN has remained the most common subtype. Notably, the proportion of biopsies performed for suspected non‐diabetic disease in diabetic patients that ultimately confirm diabetic nephropathy has increased significantly, rising from 1.4% to 10.2% over the last 20 years. Additionally, there has been a significant increase in cases of PIGN [24]. Hypertensive nephrosclerosis, amyloidosis, and vasculitis are reported less frequently. Amyloidosis is noted to be less common in Saudi Arabia compared to neighboring Arab countries.

Worth noting, mean serum creatinine at biopsy declined over time (AlFaadhel et al. 190 μmol/L; Alhasan et al. 91 μmol/L), reflecting earlier detection and prompt initiation of diagnostic evaluations.

### 3.6. Clinical Outcomes of GD

Outcome data are limited in most of the included studies. Generally, GN in adults tends to follow an aggressive course, with a higher percentage of glomeruli being affected in cases such as anti‐GBM disease, compared to immune‐complex or pauci‐immune GN. Early diagnosis and treatment consistently show improved outcomes across various types of GN. Progression to ESRD was observed in 25%–35% of crescentic and FSGS cases during follow‐up (Mitwalli et al. [14]; Mosaad et al. [21]), particularly in steroid‐resistant cases. Diseases such as anti‐GBM and ANCA‐associated types often carry a poor prognosis unless treated early and aggressively. A high serum creatinine level (> 400 μmol/L) at presentation has been strongly associated with progression to ESRD.

In contrast, certain types of GD tend to have more favorable outcomes. In children with RPGN, the prognosis is generally favorable, particularly when PIGN is the underlying cause, with 68.4% achieving complete or partial recovery [21]. MCD also has a good prognosis, especially in children, as it is typically steroid‐responsive. LN, however, shows variable outcomes depending on the class, with class IV having a worse prognosis compared to class II or V.

## 4. Discussion

GDs constitute a leading non‐diabetic cause of CKD worldwide. Kidney failure imposes the heaviest economic and health burden, while also being associated with the poorest reported quality of life among patients. Within the Middle East, Saudi Arabia ranks second in the prevalence of kidney failure among treated patients, with GDs accounting for 66.7% of incident ESKD cases [25, 26]. This systematic review offers a comprehensive overview on GDs in Saudi Arabia from 12 renal biopsy‐based studies spanning three decades. Our findings highlight important clinical and pathological patterns, along with notable regional and over‐time variations.

The remarkable consistency of the findings across different institutions, regions, and time periods underscores the robust nature of the epidemiological patterns. On the other hand, the pathology and outcomes are generally comparable to global patterns, though genetic, environmental, and healthcare system differences may explain some deviations.

FSGS remains the most prevalent primary GD in most studies conducted across various regions of the Kingdom. This high prevalence in Saudi Arabia aligns closely with patterns observed in North American populations [7]. Significant proportion of FSGS cases in Saudi Arabia may represent secondary forms related to obesity‐induced glomerulopathy, a trend that has become increasingly recognized in developed countries experiencing rising obesity rates [27]. Similarly, the clinical presentation of FSGS in Saudi Arabian patients demonstrates characteristic features that align with global descriptions of this condition. Patients with FSGS typically present with a high incidence of hypertension (86.7%), nephrotic syndrome (61.7%), hematuria (48.8%), and renal impairment (33.3%) [14].

Longitudinal data from Saudi Arabia reveal a progressive increase of IgAN, the second rank, frequency over the past two decades. However, its prevalence remains significantly lower than the rates observed in East Asian populations [8, 10]. The reasons for this increasing prevalence are not entirely clear, but potential contributing factors include improved diagnostic techniques and possible environmental or dietary changes. Previous research has suggested that changes in gut microbiota may play a role in the development of IgAN [28]. The increasing consumption of fast food and Western dietary patterns among the Saudi population may influence gut microbiota profiles and contribute to the rising prevalence of IgAN. Furthermore, pollutants, such as fine particulate matter (PM2.5) and other environmental toxins, may contribute to immune dysregulation, potentially triggering or exacerbating the disease [29]. This should be interpreted with caution as the observed temporal changes may reflect evolving diagnostic practices, variable availability of immunofluorescence, and electron microscopy, changing referral patterns.

Conversely, MPGN demonstrates interesting temporal trends in Saudi Arabia, with recent studies showing a declining prevalence over the past two decades [10]. This decline mirrors global trends observed in the United States and Asia and may be attributed to improved control of infectious diseases, widespread hepatitis vaccination, changes in environmental exposures, and evolving diagnostic criteria [7, 8].

The association between secondary GDs and various systemic conditions in Saudi Arabia reflects the broader health profile of the population and the distribution of the underlying comorbidities. The high prevalence of diabetes mellitus, hypertension, and obesity creates a substrate for metabolic kidney disease, while the genetic diversity of the Saudi population contributes to varying susceptibilities to autoimmune and hereditary conditions [30]. The spectrum of secondary GDs in Saudi Arabia is dominated by LN, which consistently emerges as the most common secondary cause of GN across multiple studies. This correlates with the increased susceptibility of certain populations to systemic lupus erythematosus, particularly those of Middle Eastern and North African descent [31]. Nevertheless, the declining trend of LN among systemic lupus erythematosus patients may reflect advancements in management, earlier intervention to prevent kidney involvement [32], or changes in the population demographics of patients undergoing kidney biopsy.

Diabetic nephropathy has become a significant cause of secondary GD in Saudi Arabia. Over the studied time period, its prevalence has increased more than seven‐fold. This rise corresponds to the dramatic increase in diabetes mellitus prevalence. The Kingdom ranks among the top 10 countries globally in terms of diabetes prevalence, making diabetic nephropathy a major public health concern with significant implications for healthcare resource allocation and CKD management [33]. Importantly, diabetic patients may also develop non‐diabetic kidney diseases, and kidney biopsy is sometimes necessary to distinguish diabetic nephropathy from other forms of GD, particularly when the clinical presentation is atypical or when diabetic retinopathy is absent [34].

### 4.1. Clinical and Public Health Implications

The high disease burden among young and middle‐aged adults carries significant socioeconomic implications and justifies investment in patient education, early diagnosis, and access to immunosuppressive therapies. High FSGS prevalence reinforces the need for steroid‐sparing agents, obesity management, and prompt genetic testing once available. Correspondingly, high MGN prevalence may warrant hepatitis B and C screening [24].

The prioritization of lupus and diabetes management within nephrology services highlights the importance of multidisciplinary care for patients at risk of glomerular injury. National diabetes prevention and control initiatives are essential to reduce the risk of renal complications. Additionally, lifestyle modifications that strengthen mucosal immunity, along with wide‐ranging efforts to improve air quality and reduce pollution, could help curb the rise in IgAN.

Given the overlap in clinical presentations, kidney biopsy is essential for differentiating FSGS, MGN, and lupus nephritis. Early recognition and standardized biopsy protocols, including IF and EM, are essential for accurate diagnosis and treatment [35]. In the current study, the included early series lacked routine IF/EM, leading to probable under‐recognition of IgAN and MPGN misclassification. All post‐2005 studies used full IF, while EM remained limited (< 25% of cases) outside major academic hospitals. Rising IgAN and MN necessitate routine IF and consideration of M‐type phospholipase‐A2 receptor antibodies in serological work‐up [36].

A national renal biopsy registry using standardized data collection would provide high‐quality evidence to enable systematic surveillance, benchmarking, and research collaboration. Furthermore, studies are needed to investigate the prevalence of genetic risk factors (e.g., APOL1, PLA2R) as well as the impact of environmental and lifestyle factors on the development of GD in the Saudi population. Lastly, research must evolve beyond prevalence to focus on long‐term outcomes, including response to therapy and progression to ESRD for different GDs.

### 4.2. Strengths and Limitations

This review applied stringent inclusion criteria to provide a comprehensive synthesis of decades of regional biopsy data and patterns over time. However, it is constrained by the limitations of the original studies. Significant heterogeneity in study methodologies, diagnostic criteria, and reporting standards complicates direct comparisons. Moreover, the reliance on retrospective data from tertiary referral centers may introduce selection bias, as these centers inherently manage more severe or diagnostically challenging cases. Some cohorts included in this analysis were limited in size, warranting caution in the interpretation of these specific subgroups [19, 21, 22]. Most importantly, the absence of a national kidney biopsy registry means these synthesized data may not fully reflect the true prevalence of GDs and limit incidence inference.

## 5. Conclusion

GDs pose a significant healthcare burden in Saudi Arabia, with FSGS being the most prevalent primary GD and lupus nephritis the most prevalent secondary GD. Temporal trends indicate a rise in diabetic nephropathy, driven by increasing diabetes prevalence and autoimmune susceptibility. There is also an increase in IgA nephropathy, possibly linked to environmental and dietary changes, alongside a decline in membranoproliferative glomerulonephritis.

The findings highlight the need for early diagnosis, standardized biopsy protocols, and multidisciplinary management of systemic conditions like diabetes and lupus. A national renal biopsy registry would improve surveillance and research collaboration. Future studies should focus on genetic risk factors, long‐term outcomes, and therapeutic responses to optimize patient care.

**Nomenclature**
ANCA: Antineutrophil cytoplasmic antibody
CKD: Chronic kidney disease
EM: Electron microscopy
ESKD: End‐stage kidney disease
FSGS: Focal segmental glomerulosclerosis
GD: Glomerular disease
GN: Glomerulonephritis
IF: Immunofluorescence
IgAN: IgA nephropathy
KDIGO: Kidney disease improving global outcomes
LN: Lupus nephritis
MCD: Minimal change disease
MPGN: Membranoproliferative glomerulonephritis
PIGN: Post‐infectious glomerulonephritis
PRISMA: Preferred Reporting Items for Systematic Reviews and Meta‐Analyses
RPGN: Rapidly progressive glomerulonephritis

## Author Contributions

All authors contributed to the conceptualization of the article, performed the literature search and data extraction, and participated in drafting and critically revising the work.

## Funding

No funding was received for this manuscript.

## Ethics Statement

The authors have nothing to report.

## Consent

The authors have nothing to report.

## Conflicts of Interest

The authors declare no conflicts of interest.

## Supporting Information

The supporting information document includes two tables:

Table S1: Keywords used for the search strategy.

Table S2: Newcastle–Ottawa Scale quality assessment of included studies.

## Supporting information


**Supporting Information** Additional supporting information can be found online in the Supporting Information section.

## Data Availability

All data analyzed in this study were extracted from original published articles and are publicly available.
