# Supplementary material for: The Spectrum and Temporal Trends of Glomerular Diseases in Saudi Arabia: A Systematic Review of Biopsy‐Based Studies
Source: Int J Nephrol. 2026 Apr 23;2026:3670670. doi: 10.1155/ijne/3670670 (PMC13106946; doi:10.1155/ijne/3670670)
Supplement: Supplementary file 1 — Supporting Information Additional supporting information can be found online in the Supporting Information section. [file IJNE-2026-3670670-s001.docx]

**Supplementary materials**

**Table S1 Keywords used for search strategy**

| **Category** | **Keywords and MeSH terms** |
| --- | --- |
| **Glomerular Diseases** | "glomerulonephritis", "glomerular disease", "chronic kidney disease", "IgA nephropathy", "nephrotic syndrome", "focal segmental glomerulosclerosis", "membranous nephropathy" |
| **Prevalence** | "epidemiology", "prevalence", "incidence", "rate", "frequency", "disease burden", "population-based study", "cohort study", "cross-sectional study" |
| **Saudi Arabia** | "Saudi Arabia", "Middle East", "Arab population", "Gulf region", "KSA" |
| **Diagnostic Approaches** | "renal biopsy", "immunofluorescence", "light microscopy", "electron microscopy", "histopathology" |
| **Risk Factors** | "diabetes mellitus", "hypertension", "autoimmune diseases", "genetic predisposition" |

**Table S2 Newcastle-Ottawa scale quality assessment of included studies**

| **Study (Author, Year)** | **Design** | **Selection (max 4)** | **Comparability (max 2)** | **Outcome (max 3)** | **Total Stars (out of 9)** | **Quality** | **Notes/Limitations** |
| --- | --- | --- | --- | --- | --- | --- | --- |
| Mitwalli et al., 1996 | Retrospective | ★★★★ | ★★ | ★★ | 8 | High | Single-center, some retrospective bias |
| Huraib et al., 2000 | Registry, Multicenter | ★★★★ | ★★ | ★★ | 8 | High | National multicenter, large sample, standardized forms |
| Shawarby et al., 2010 | Retrospective | ★★★★ | ★★ | ★★ | 8 | High | Long period, limited EM/IF in some cases |
| Nawaz et al., 2013 | Retrospective | ★★★★ | ★★ | ★★ | 8 | High | Single-center, clear inclusion criteria |
| Mokhtar et al., 2014 | Retrospective | ★★★ | ★★ | ★★ | 7 | High | MesPGN focused, some referral bias, limited clinical follow-up |
| Al-Hussain et al., 2017 | Retrospective | ★★★ | ★★ | ★★ | 7 | High | Focused on crescentic GN, robust pathology |
| AlMatham et al., 2017 | Retrospective | ★★★★ | ★★ | ★★ | 8 | High | Good population coverage, robust data, some missing data |
| Mosaad et al., 2018 | Retrospective | ★★★ | ★ | ★★ | 6 | Moderate | Small sample, single-center, limited follow-up, rare outcome |
| Manzoor et al., 2019 | Retrospective | ★★★ | ★ | ★★ | 6 | Moderate | Small sample, single-center, limited EM, possible selection bias |
| AlFaadhel et al., 2019 | Registry, Multicenter | ★★★★ | ★★ | ★★ | 8 | High | Multicenter, large sample, robust temporal analysis |
| Alhasan et al., 2020 | Retrospective | ★★★★ | ★★ | ★★ | 8 | High | Pediatric, multicenter, standardized pathology, some retrospective bias |
| Jalalah, 2009/2020 | Retrospective | ★★★★ | ★★ | ★★ | 8 | High | Largest Western region series, robust pathology |
